# Rosa roxburghii Tratt juice ameliorates metabolic disorder in arsenicosis patients based on the analysis of untargeted plasma metabolomics

**DOI:** 10.3389/fphar.2025.1488113

**Published:** 2025-04-22

**Authors:** Luming Yang, Kai Zhu, Wenjuan Wang, Baofei Sun, Peng Luo, Aihua Zhang

**Affiliations:** The Key Laboratory of Environmental Pollution Monitoring and Disease Control, Ministry of Education, Department of Toxicology, School of Public Health, Guizhou Medical University, Guiyang, Guizhou, China

**Keywords:** arsenic, arsenicosis patients, metabolic disorder, Rosa roxburghii Tratt juice, untargeted metabolomics

## Abstract

Arsenic is an environmental metalloid contaminant known to induce multi-system and multi-organ damage, yet the precise toxicological mechanisms remain unclear. Moreover, effective low-toxicity interventions or treatments are lacking. This study aims to investigate the potential ameliorative effects of Rosa roxburghii Tratt juice (RRTJ) on metabolic disorders in arsenicosis patients, with a focus on plasma metabolite profiles. Using ultra-high-performance liquid chromatography-tandem mass spectrometry (UHPLC-MS/MS), we analyzed the plasma metabolic profiles of arsenicosis patients before and after RRTJ intervention. After RRTJ intervention, significant alterations were observed in the plasma levels of 61 metabolites, with 30 metabolites upregulated and 31 downregulated. These metabolites were predominantly involved in six key biological pathways, including taurine and hypotaurine metabolism, histidine metabolism, β-alanine metabolism, glycine, serine, and threonine metabolism, pentose and glucuronate interconversions, as well as cysteine and methionine metabolism. In conclusion, RRTJ intervention may effectively alleviate metabolic disorders associated with arsenic toxicity, potentially through its antioxidant, anti-inflammatory effects and regulation of methylation pathways.

## Graphical Abstract

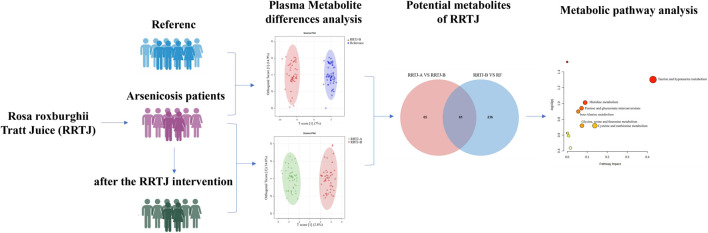

## 1 Introduction

Arsenic is an environmental metalloid contaminant that is widely present in water, food, and the environment (Kang et al., 2011). Ingestion of excessive arsenic can lead to its accumulation in vital organs such as the lungs, liver, kidneys, and skin, causing a variety of diseases, including cardiovascular diseases (Domingo-Relloso et al., 2022), disorders of the nervous system (Thakur et al., 2021), ailments affecting the digestive system (Zhong et al., 2021) and respiratory conditions (Wang W. et al., 2020). Research has demonstrated that the pathogenesis of systemic damage induced by arsenic primarily encompasses immune dysregulation, oxidative stress, epigenetic alterations and metabolic disturbances (Rahaman et al., 2021; Jamal et al., 2020; Chakraborty et al., 2022).

In recent years, food and drug dual-use substances have been widely recognized for their ability to regulate metabolic activities and alleviate disease processes (Khan et al., 2022; Bhambri et al., 2022). Rosa roxburghii Tratt (RRT), a fruit species endemic to Guizhou Province, is known for its rich nutrients and bioactive compounds, including vitamin C, flavonoids, polyphenolic compounds and triterpenoids (Xu et al., 2022). The presence of these natural constituents endows RRT with exceptional nutritional value and potential health benefits (Huang et al., 2022). We have previously demonstrated that the juice derived from freshly squeezed Rosa roxburghii Tratt exhibits immunoregulatory potential by significantly reducing the population of Th17 cells and modulating the Th17-related inflammatory cytokines IL-17 and IL-6 (Dong et al., 2022). Rosa roxburghii Tratt juice (RRTJ) can also reverse elemental imbalances caused by arsenic, such as elevated levels of Al, As, and Fe, and decreased levels of Cu, Zn, Se, etc., thereby regulating the body’s elemental balance (Xu et al., 2022). *In vivo* studies have demonstrated that RRTJ effectively counteracts liver oxidative damage in a rat model of arsenic poisoning by inhibiting the acetylation of histone H3K18 in the liver (Ma et al., 2023). Moreover, RRTJ polyphenols may indirectly mitigate lung injury by reducingphenylalanine levels and increasing tryptophan levels (Tang et al., 2022). Despite the various potential disease-alleviating mechanisms demonstrated by Rosa roxburghii Tratt preparations in in vitro experiments and animal models, the capacity of these preparations to modulate systemic health damage in arsenicosis patients remains to be explored through further epidemiological studies and omics analysis.

With the rapid development of omics technology, metabolomics has been extensively applied in various domains, including disease diagnosis, drug toxicity assessment, nutritional interventions and drug mechanism research, due to its high resolution, high sensitivity, and high throughput capabilities (Rispoli et al., 2021). Metabolomics is the scientific investigation of metabolites produced through endogenous reactions within the body, which closely reflect disease phenotypes. Most biological processes such as cell signaling, energy transfer and intercellular communication are complexly regulated by metabolites. Under specific physiological and pathological conditions, metabolites can serve as distinctive molecular markers while offering a comprehensive perspective on endogenous fluctuations in response to exogenous stimuli (Wang R. et al., 2020). Plasma metabolites can reflect changes in metabolic pathways associated with endogenous metabolites in a system, organ or whole organism. By detecting variations in plasma metabolites, a more precise understanding of the relationship between disease aetiology, drugs and disease development can be achieved (Qiu et al., 2023). Untargeted metabolomics can detect hundreds of small-molecule metabolites to find sensitive markers of disease. In toxicology research, the use of untargeted metabolomics technology can more quickly and effectively screen the targets of exogenous chemicals so as to adopt targeted treatment measures (Szeremeta et al., 2021). Our previous research has shown that arsenicosis patients exhibit metabolic disorders, which are associated with the severity of arsenic poisoning symptoms (Zhang et al., 2023). However, the potential of RRTJ to reverse the metabolic profile of arsenicosis patients remains uncertain. Therefore, this study employs untargeted metabolomics techniques to assess the impact of RRTJ on plasma metabolites in arsenicosis patients, aiming to provide a reference for subsequent mechanistic investigations.

To investigate the impact of RRTJ on metabolic disorders in arsenicosis patients, we initially conducted a comprehensive analysis of the plasma metabolic profiles of arsenicosis patients before and after an RRTJ intervention. Subsequently, we further scrutinized the differential metabolites before and after the RRTJ intervention to elucidate the influence of RRTJ on plasma metabolites. The ultimate goal of this research was to provide scientific evidence from a metabolomics perspective to support the potential application value of RRTJ in alleviating arsenic poisoning.

## 2 Materials and methods

### 2.1 Study design

A total of 46 arsenicosis patients (arsenicosis patients before the RRTJ intervention group, RRTJ-B) in Yuzhang Town, Xingyi City, Guizhou Province, China, participated in this study. Inclusion criteria: (1) all participants were permanent residents of Jiaole village; (2) have a history of arsenic exposure in coal-burning arsenic area; (3) were diagnosed with arsenicosis according to the Endemic Arsenic Poisoning Diagnostic Criteria (WS/T 211–2015). Exclusion criteria: (1) a history of occupational arsenic exposure; (2) a heritage metabolic disease or inborn metabolic deficiency; (3) a recent history of drugs that could affect metabolic function (Medications for Hypertension, Diabetes, and Cardiovascular Diseases). (4) individuals who themselves or their family members were allergic to RRTJ. Additionally, A total of 60 healthy villagers were randomly enrolled from Shang Batian village, an arsenic-free area, to form the reference group (RF). The reference group shared similar dietary habits, economic status, nutritional status, and lifestyle as the arsenicosis group, but did not have a history of using arsenic-rich coal or any signs of arsenic poisoning. Met the exclusion criteria for the arsenicosis group.

**FIGURE 1:**
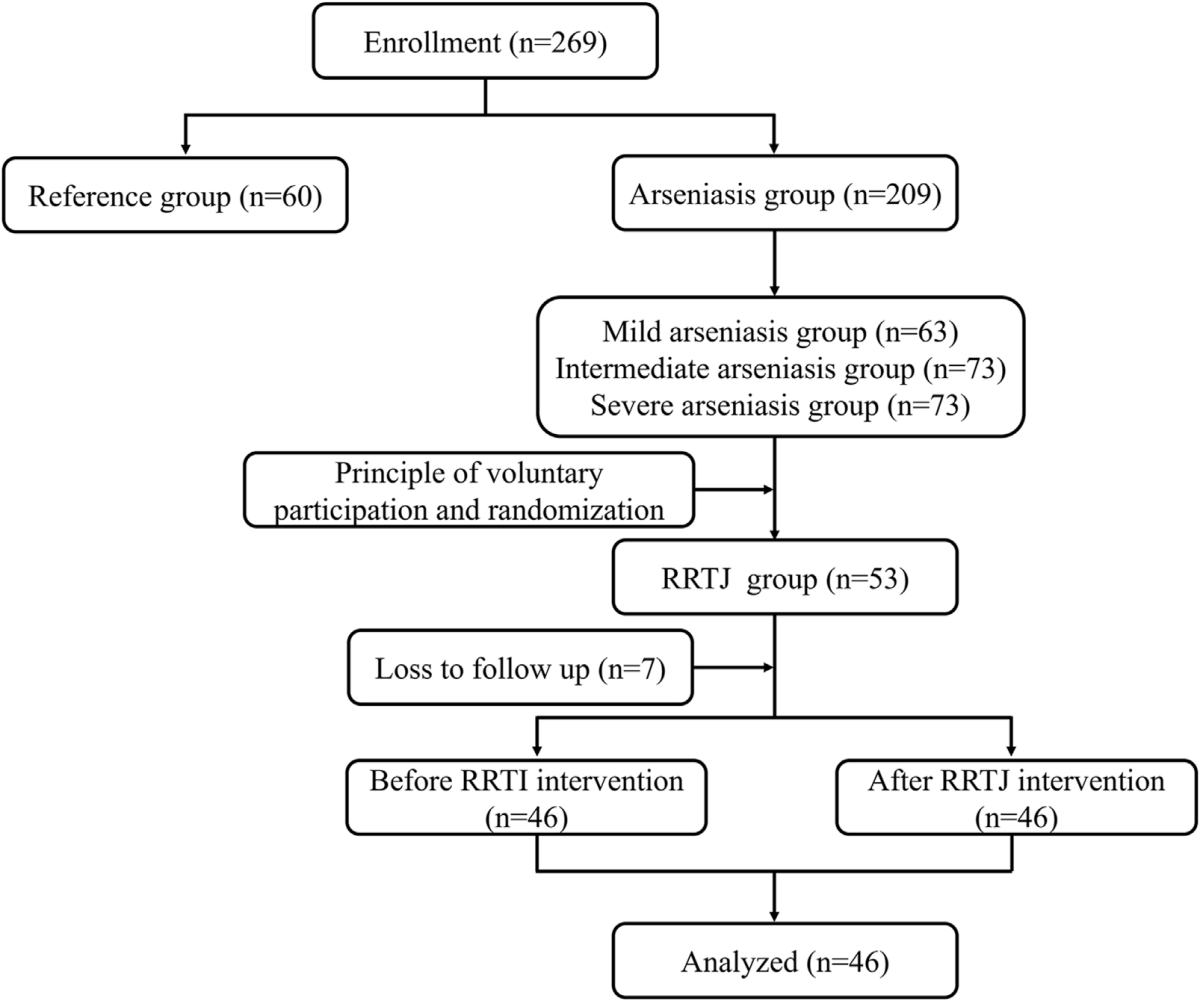
Grouping and study-flow diagram.

RRTJ was purchased from Sinopharm Group Guizhou Health Industry Development Co., LTD., National Health Commission, PRC Health Food License No. [2002]0004. RF did not receive any intervention measures. Arsenicosis patients were given 20 mL RRTJ per day (arsenicosis patients after the RRTJ intervention group, RRTJ-A), according to the recommended dosage in the health food instructions, for 3 months (Xu et al., 2022) (Figure 1).

Peripheral blood was collected into vacuum tubes containing ethylenediamine tetraacetic acid (EDTA). The tubes were centrifuged immediately, and the plasma was separated from the other blood components. The plasma was then dispensed and stored at −80°C.

Ethical approval for this study was obtained from the Ethics Committee of Guizhou Medical University (No. 201403001). Written informed consent was received from all participants before experimentation.

### 2.2 Metabolomics processing

The metabolomics analysis utilized the Agilent 1,290 Infinity LC system (Agilent Technologies, United States) in conjunction with the AB SCIEX Triple TOF 6600 system (AB SCIEX, United States), interfaced with an electrospray ionization source, and followed a non-targeted metabolomics protocol based on established procedures. Blood plasma samples were preprocessed by combining them with an equal volume of cold methanol/acetonitrile/water (2:2:1), then centrifuged at 14,000 g and 4°C for 15 min. Each quality control (QC) sample was assessed for measurement stability and reproducibility using a volume of 10 μL. Chromatographic separation occurred on an ACQUITY UPLC BEH column (2.1 × 100 mm, 2.1 μm particle size, Waters, United States). Prior to injection onto the column, blood plasma samples were dissolved in a mixture of methanol and ethanol (1:1) at a volume of 100 μL. The mobile phase consisted of water containing ammonium acetate and ammonia along with acetonitrile running at a flow rate of500 μL/min in a gradient elution program. Gas pressures for elution were set as follows: gas-1:60 psi; gas-2:60 psi; curtain gas:30 psi; spray voltages used for positive and negative modes were +5.5 kV and −5.5 kV respectively; source temperature maintained at600°C; precursor ion mass-to-charge ratio range from MS1 was between60-1,000 Da while product ions from MS2 ranged between25-1,000 Da; collision energy applied was35 ± 15 eV; desolvation voltage utilizedwas60 V.A window width exclusionof4Dafor isotopeswas also implemented. Once mass spectra measurementswere obtained, XCMS softwarewas employedfor metabolite extractionand annotation. The peak selection process involvedthe useofCentWave algorithm,and Metabolite Profile Annotationalgorithm suite (CAMERA)was utilizedfor annotatingisotopesand complexes.

### 2.3 Statistical analyses

The data were statistically analysed using MetaboAnalyst. *T*-tests and fold change (FC) analysis were used to compare two groups. Metabolites with a variable importance in projection (VIP) > 1.0, *P* < 0.05 and FC > 1.5 or FC < 0.67 (Zhu et al., 2022; Wang et al., 2023) were selected as potential biomarkers. MetaboAnalyst was also used for principal component analysis (PCA), partial least squares-discriminant analysis (PLS-DA), orthogonal partial least squares discriminant analysis (OPLS-DA) and heat map analysis to reflect the changes in each metabolite. Metabolic pathway analysis was performed using the Kyoto Encyclopaedia of Genes and Genomes (KEGG) database.

The general participant data were analysed using SPSS 22.0 software. Graphs were plotted using GraphPad Prism software, version 9.0. Quantitative data were tested for normality and homogeneity of variance. Normally distributed data are presented as the mean ± standard deviation (SD), and independent samples *t*-tests were used for two-group comparisons. The Chi-square test was used to compare rates between groups. Differences were considered statistically significant at *P* < 0.05.

## 3 Results

### 3.1 Demographic data of the study population

The sample comprised 106 participants, including 46 arsenicosis patients and 60 reference individuals. The reference residents and arsenicosis patients exhibited similar characteristics in terms of age, gender, as well as smoking and alcohol consumption history, indicating a fundamental consistency in the demographic structure and lifestyle among between the two groups (all *P* > 0.05, Table 1).

**TABLE 1:** General information of observed subjects.

Variable	Reference (n = 60)	Arseniasis (n = 46)	Statistical value	*P* Value
Sex (N, %)				
Male	25 (41.67)	21 (45.65)	0.17^b^	0.68
Female	35 (58.33)	25 (54.35)		
Age (mean ± SD, years)	56.92 ± 8.79	52.85 ± 7.50	2.86^a^	0.09
Smoking (N, %)				
Never Smoking	36 (0.60)	28 (0.61)	0.08^b^	0.93
Smoking	24 (0.40)	18 (0.39)		
Alcohol Drinking (N, %)				
Never Drinking	46 (0.77)	31 (0.67)	1.13^b^	0.29
Drinking	14 (0.23)	15 (0.33)		

^a^ *t*-test, the statistical value is *t* value.

^b^ Chi-square test, the statistical value is *χ*2 value.

### 3.2 RRTJ ameliorated plasma metabolic spectrum disorders in arsenicosis patients

**FIGURE 2:**
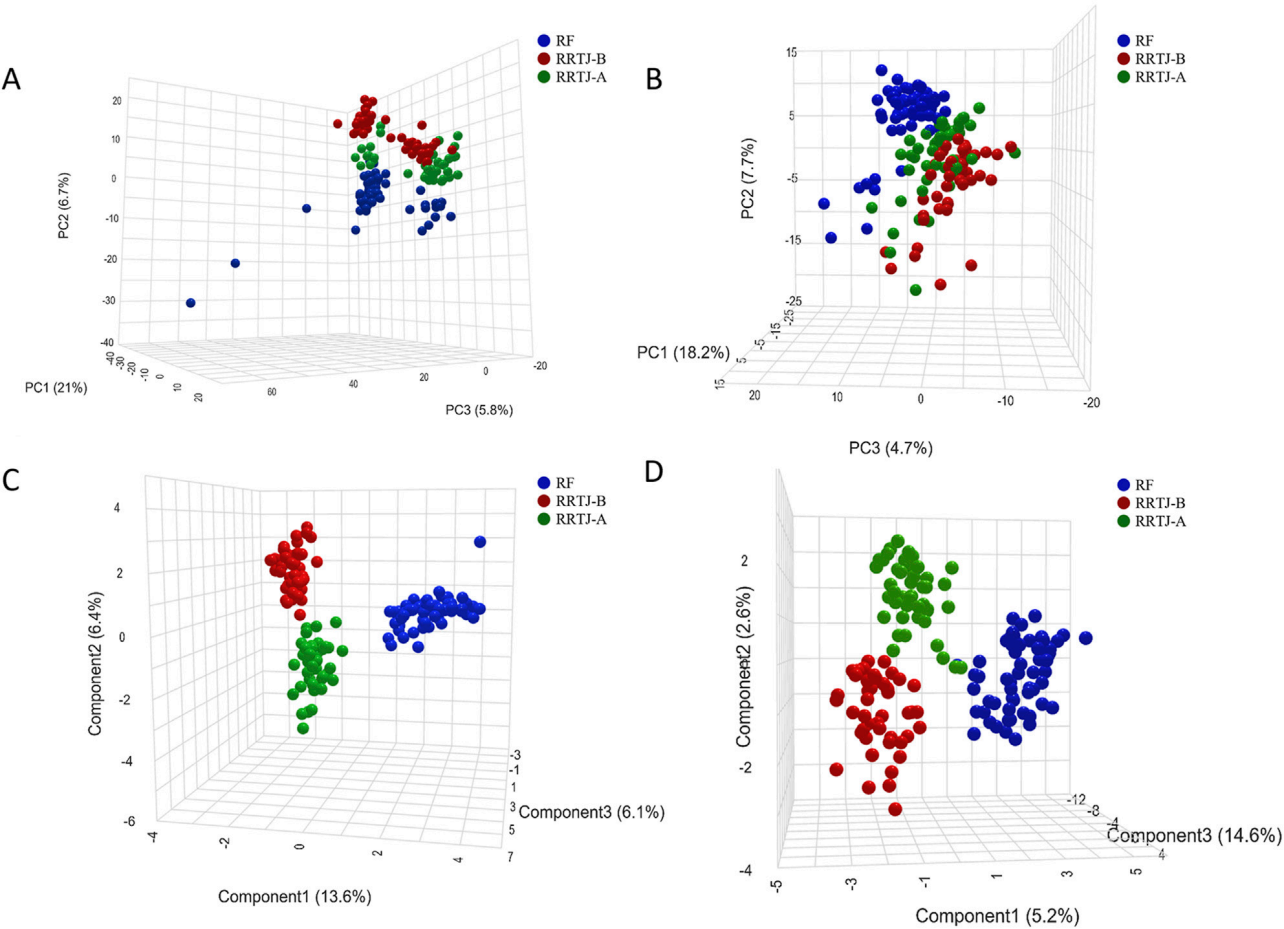
Metabolite changes in the plasma across groups were analyzed using PCA **(A,B)** and PLS-DA **(C,D)** score plot.

To investigate the effect of RRTJ on the metabolics of arsenicosis patients, we comprehensively analysed the plasma metabolic profiles and metabolites of the RF, RRTJ-B and RRTJ-A groups using ultra-high performance liquid chromatography-tandem mass spectrometer (UHPLC-MS/MS). A total of 1,264 metabolites were identified in the plasma samples, with 807 detected in positive ion mode and 457 detected in negative ion mode. Subsequently, PCA score plots were employed to examine the differences between the RF, RRTJ-B and RRTJ-A groups. The PCA plots are shown in Figures 2A,B. The separation between the RF and RRTJ-B groups was significant in the PCA score plot, indicating that the plasma metabolites of the arsenicosis patients had changed significantly. In addition, the samples in the RRTJ-A group were closer to those in the RF group, indicating that RRTJ reversed the metabolic disorder of arsenicosis patients to some extent.

To further investigate the effect of RRTJ on the plasma metabolic profile of arsenicosis patients, a supervised partial least squares discriminant analysis (PLS-DA) model was applied. The PLS-DA score plots (Figures 2C,D) showed clear separation among the RF, RRTJ-B and RRTJ-A groups, and clustered distinctly in both the positive and negative ion modes, indicating that the group differences were more remarkable than the individual differences. The plasma metabolic profile of the arsenicosis patients after the RRTJ intervention was significantly separated from that of the arsenicosis patients before the RRTJ intervention, indicating that RRTJ affected the metabolic profile of arsenicosis patients and improved the endogenous metabolite disorder. These findings highlight the substantial alterations in the plasma metabolic profile of arsenicosis patients following an RRTJ intervention.

### 3.3 RRTJ restored multiple plasma metabolite levels in arsenicosis patients

**FIGURE 3:**
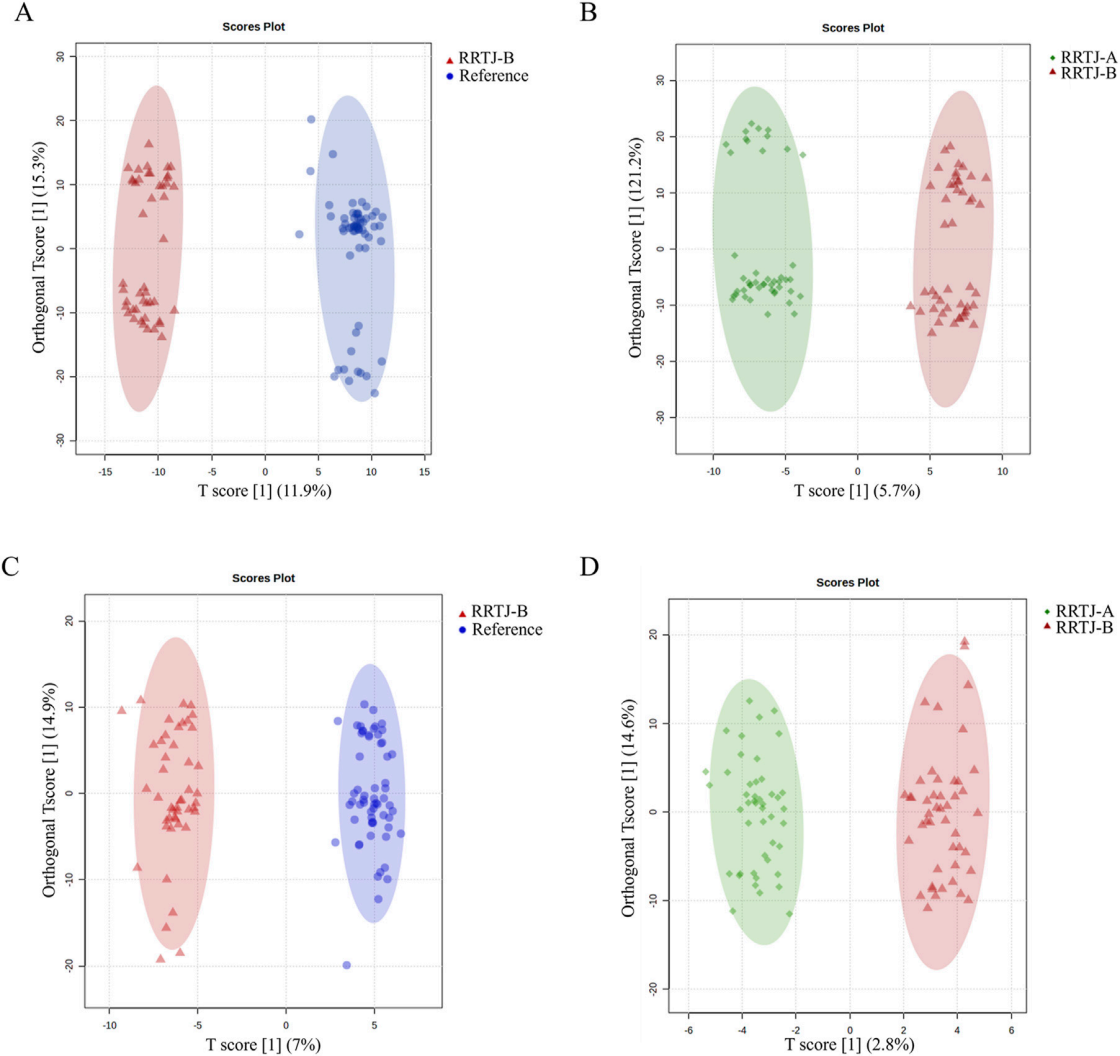
The changes in plasma metabolites among groups were analyzed using OPLS‐DA in both positive ion mode** (A,B)** and negative ion mode **(C,D)**.

To reveal the effect of RRTJ on metabolites in arsenicosis patients, supervised multivariate analysis was performed to further compare the plasma metabolites of the RF and RRTJ-B groups, as well as the RRTJ-B and RRTJ-A groups. OPLS-DA is a supervised method of multivariate analysis. As shown in Figure 3, OPLS-DA revealed good separation of the RF and RRTJ-B groups in both positive and negative ion modes. Similarly, the RRTJ-A group was clearly separated from the RRTJ-B group. This verifies the stability of the OPLS-DA model. These data suggest that the between-group differences are much larger than the within-group differences.

**FIGURE 4:**
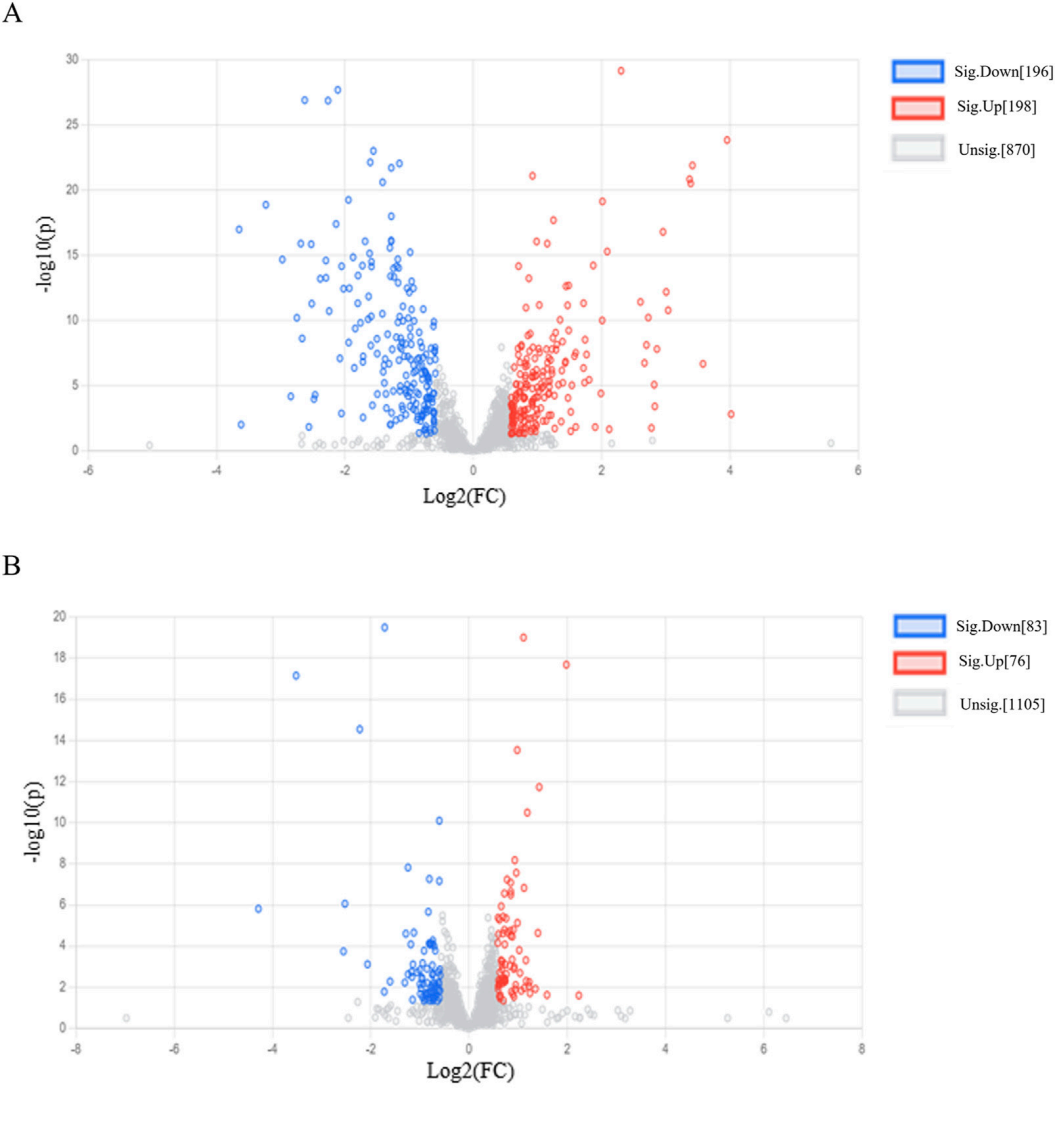
Volcanic plots of plasma metabolite.** (A)** RRTJ-B group vs. RF group, **(B)** RRTJ-A group vs. RRTJ-B group.

A VIP>1 indicates that a metabolite contributes significantly to the differentiation between the two groups and serve as a potential metabolite biomarker of RRTJ. In addition, based on previous research, *P* < 0.05, FC > 1.5 or FC < 0.67 were also used to identify potential metabolites of RRTJ to alleviate arsenic toxicity (Fu et al., 2023; Zhou et al., 2023). The volcano plot (Figure 4A) revealed 394 differential metabolites between the RF and RRTJ-B groups. Among them, 196 were upregulated and 198 were downregulated. Figure 4B shows that there were 159 differential metabolites between the RRTJ-B and RRTJ-A groups. Of these, 76 were upregulated and 83 were downregulated.

**FIGURE 5:**
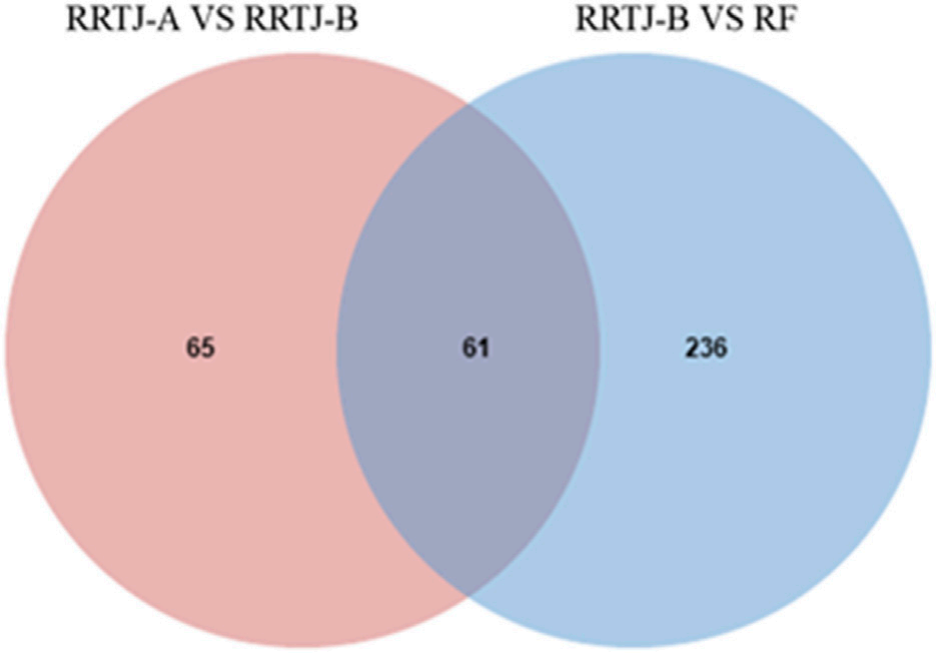
Venn diagram of differential metabolite between RRTJ-B group vs. RF group and RRTJ-A group vs. RRTJ-B group.

**FIGURE 6:**
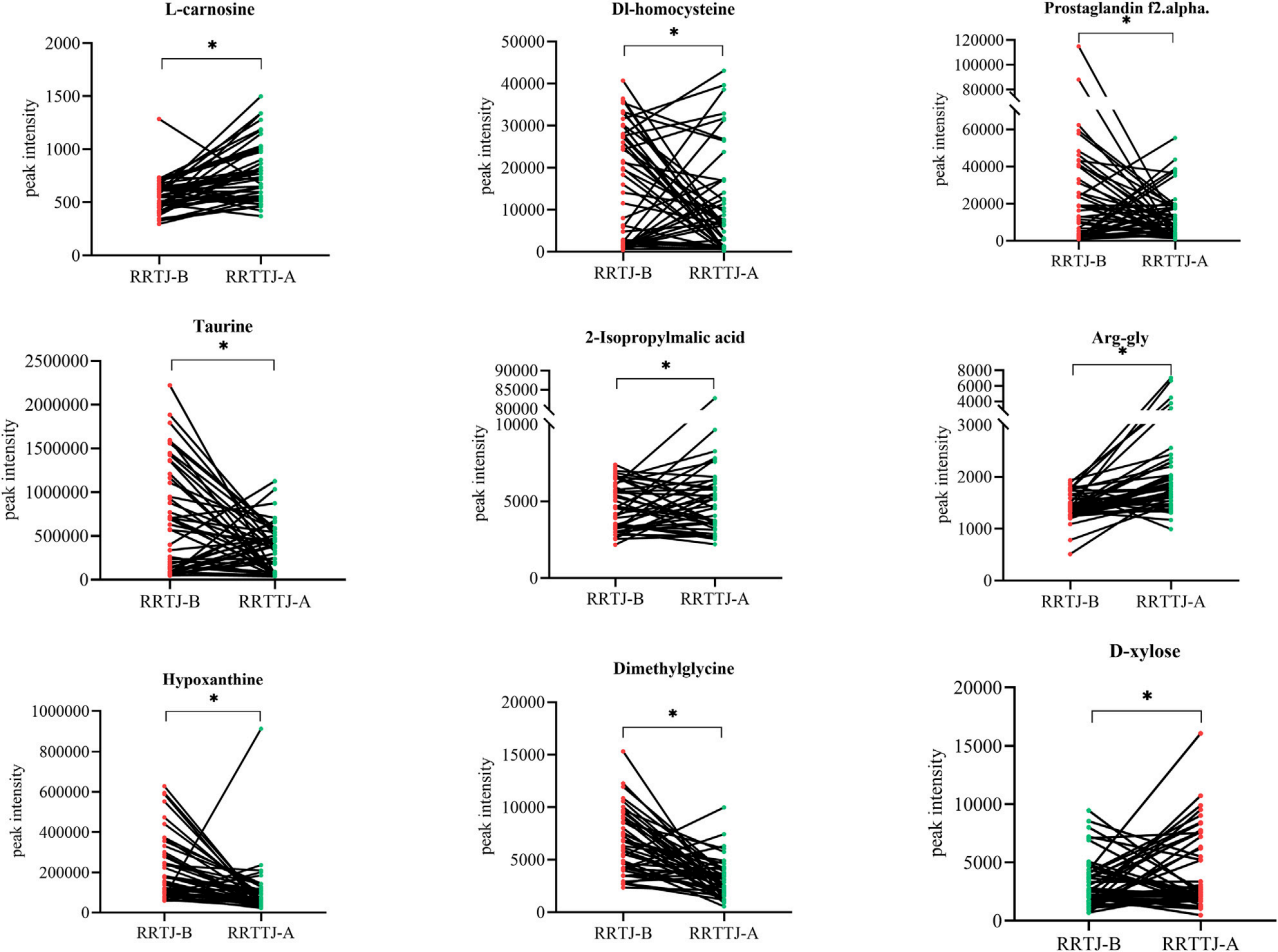
Scatter plot of peak intensity of representative differential metabolites. ^*^ *P* < 0.05.

**FIGURE 7:**
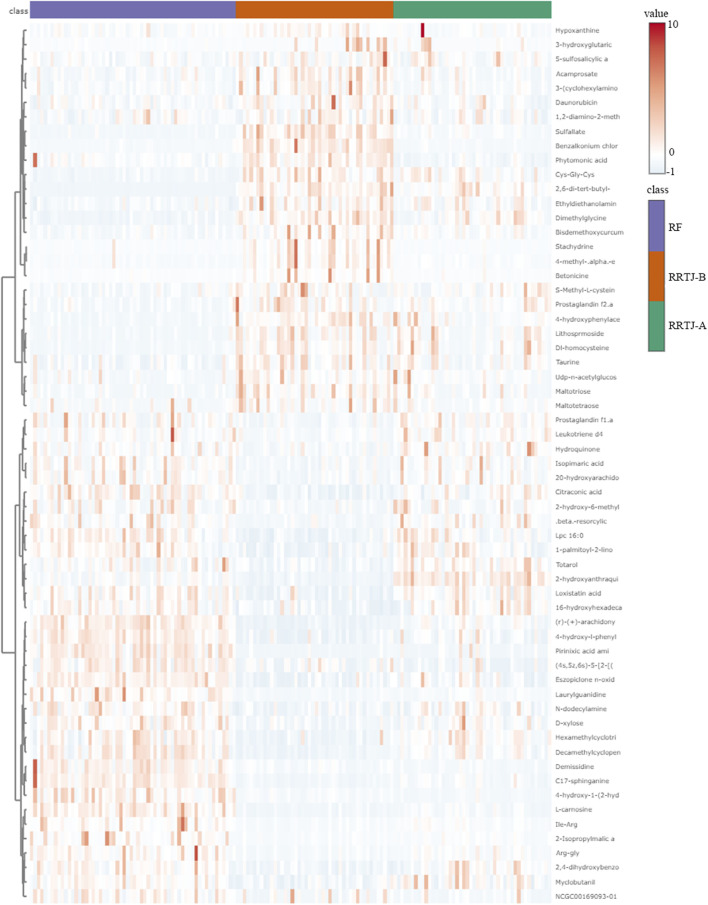
Heat map of differential plasma metabolites.

A Venn diagram was further constructed to identify intersecting metabolites. As shown in the Venn diagram (Figure 5), a total of 61 metabolites were significantly modulated by RRTJ (Table 2). Among them, 30 metabolites, including L-carnosine, 2-isopropylmalic acid, arg-gly and D-xylose, were upregulated (*P* < 0.05), and 31 metabolites, including dimethylglycine, taurine, Dl-homocysteine, hypoxanthine and prostaglandin f2. α, were downregulated in the RRTJ-A group compared to the RRTJ-B group (*P* < 0.05, Figure 6). To simultaneously visualize the contents of all potential targeted metabolites in the different groups, a heat map was constructed (Figure 7). In this heat map, red to blue represents a reduced metabolite content. The heatmap results indicated that RRTJ significantly reversed the abnormal levels of 61 metabolites and returned their expression levels to normal.

**TABLE 2:** Potential biomarkers associated with RRTJ intervention.

Compound name	m/z	rt(s)	Adduct	VIP	Change trend	
					RRTJ-B/RF	RRTJ-A/RRTJ-B
(r)-(+)-arachidonyl-1′-hydroxy-2′-propylamide	362.32	65.13	[M + H]+	3.50	↓^*^	↑^**^
Decamethylcyclopentasiloxane	371.10	31.51	[M + H]+	3.17	↓^*^	↑^**^
Hexamethylcyclotrisiloxane	223.06	32.44	[M + H]+	2.60	↓^**^	↑^**^
L-carnosine	227.11	441.52	[M + H]+	2.38	↓^**^	↑^**^
Ile-Arg	288.20	428.92	[M + H]+	2.34	↓^**^	↑^**^
Myclobutanil	289.12	287.32	[M + H]+	2.18	↓^**^	↑^**^
Arg-gly	232.14	584.12	[M + H]+	2.16	↓^**^	↑^**^
Laurylguanidine	228.27	138.93	[M + H]+	2.13	↓^**^	↑^**^
Eszopiclone n-oxide	143.08	494.23	[M + H-C11H7ClN4O2]+	1.87	↓^**^	↑^**^
C17-sphinganine	288.29	39.47	[M + H]+	1.83	↓^**^	↑^**^
N-dodecylamine	186.22	158.76	[M + H]+	1.74	↓^**^	↑^**^
4-hydroxy-l-phenylglycine	168.08	430.05	[M + H]+	1.57	↓^**^	↑^**^
20-hydroxyarachidonic acid	321.24	83.05	[M + H]+	1.47	↓^**^	↑^*^
Isopimaric acid	303.23	82.74	[M + H]+	1.40	↓^**^	↑^*^
D-xylose	151.06	103.30	[M + H]+	1.32	↓^**^	↑^*^
2-Isopropylmalic acid	177.09	507.38	[M + H]+	1.12	↓^**^	↑^*^
Pirinixic acid aminothiazole	513.13	534.05	[M + H]+	1.04	↓^**^	↑^*^
2-hydroxyanthraquinone	223.03	34.19	[M-H]-	5.28	↓^**^	↑^**^
Totarol	285.24	58.00	[M-H]-	3.42	↓^**^	↑^**^
Loxistatin acid	269.21	54.29	[M-H-CO2]-	3.41	↓^**^	↑^**^
16-hydroxyhexadecanoic acid	271.23	79.88	[M-H]-	2.72	↓^**^	↑^**^
.beta.-resorcylic acid	153.02	26.61	[M-H]-	2.62	↓^**^	↑^**^
1-palmitoyl-2-linoleoyl-sn-glycero-3-phosphocholine	802.56	156.48	[M + HCO2]-	2.58	↓^**^	↑^**^
Lpc 16:0	540.33	223.80	[M + HCOO]-	2.56	↓^**^	↑^**^
Citraconic acid	129.02	625.41	(M-H)-	2.53	↓^**^	↑^**^
2,4-dihydroxybenzophenone	213.05	51.75	[M-H]-	2.48	↓^**^	↑^**^
Prostaglandin f1.alpha	337.24	85.67	[M-H-H2O]-	2.40	↓^**^	↑^**^
Leukotriene d4	495.26	194.48	[M-H]-	2.16	↓^*^	↑^*^
Hydroquinone	109.03	25.17	[M-H]-	1.89	↓^**^	↑^*^
2-hydroxy-6-methylquinoline-3-carbaldehyde	186.04	252.34	[M-H]-	1.44	↓^**^	↑^*^
Phytomonic acid	279.28	36.06	[M + H-H2O]+	3.38	↑^**^	↓^**^
Benzalkonium chloride (c12)	304.30	129.18	[M]+	3.32	↑^**^	↓^**^
Sulfallate	116.05	33.32	[M + H-C3H5ClS]+	2.97	↑^**^	↓^**^
4-methyl-.alpha.-ethylaminopentiophenone	202.15	323.15	[M + H-H2O]+	2.13	↑^**^	↓^**^
Stachydrine	144.10	323.15	[M + H]+	2.12	↑^**^	↓^**^
Dimethylglycine	104.07	360.96	[M + H]+	2.09	↑^**^	↓^**^
2,6-di-tert-butyl-4-hydroxymethylphenol	219.17	33.86	[M + H-H2O]+	1.85	↑^**^	↓^**^
4-hydroxy-1-(2-hydroxyethyl)-2,2,6,6-tetramethylpiperidine	202.18	92.88	[M + H]+	1.84	↑^**^	↓^**^
Betonicine	160.10	353.85	[M + H]+	1.67	↑^**^	↓^**^
1,2-diamino-2-methylpropane	72.08	363.89	[M + H-NH3]+	1.64	↑^**^	↓^**^
Bisdemethoxycurcumin	147.05	323.04	[M + H-C10H10O2]+	1.64	↑^**^	↓^**^
Ethyldiethanolamine	134.12	338.09	[M + H]+	1.56	↑^**^	↓^**^
Cys-Gly-Cys	282.06	577.71	[M + H]+	1.55	↑^**^	↓^**^
Daunorubicin	363.09	348.66	[M + H-C6H15O4N]+	1.43	↑^**^	↓^**^
Hypoxanthine	137.04	178.90	[M + H]+	1.14	↑^**^	↓^*^
Demissidine	400.38	40.91	[M + H]+	1.12	↑^**^	↓^*^
(4s,5z,6s)-5-oxy-4h-pyran-3-carboxylic acid	589.15	521.66	[M + Na]+	1.01	↑^**^	↓^*^
Acamprosate	180.03	38.54	[M-H]-	3.44	↑^**^	↓^**^
3-(cyclohexylamino)-2-hydroxy-1-propanesulfonic acid	236.09	30.99	[M-H]-	3.36	↑^**^	↓^**^
Taurine	124.01	347.75	[M-H]-	2.53	↑^**^	↓^**^
Maltotriose	503.16	591.63	[M-H]-	2.34	↑^**^	↓^**^
4-hydroxyphenylacetic acid	151.01	350.16	[M-H]-	2.30	↑^**^	↓^**^
Maltotetraose	665.21	669.70	[M-H]-	2.06	↑^*^	↓^**^
Lithosprmoside	328.10	342.91	[M-H]-	1.97	↑^**^	↓^*^
S-Methyl-L-cysteine	134.03	353.71	[M-H]-	1.87	↑^**^	↓^*^
Prostaglandin f2.alpha	309.20	200.87	[M-H-C2H4O]-	1.87	↑^**^	↓^*^
Dl-homocysteine	116.03	337.88	[M-H-H2O]-	1.70	↑^**^	↓^*^
5-sulfosalicylic acid	434.98	548.89	[2M-H]-	1.57	↑^*^	↓^*^
NCGC00169093-01	249.15	53.92	[M-H]-	1.55	↑^*^	↓^*^
3-hydroxyglutaric acid	147.04	125.95	[M-H]-	1.39	↑^**^	↓^*^
Udp-n-acetylglucosamine	606.07	591.25	[M-H]-	1.27	↑^**^	↓^*^

↑ indicates increase; ↓ indicates decrease; *P < 0.05, **P < 0.001.

### 3.4 RRTJ ameliorates metabolic disorder in arsenicosis patients through various metabolic pathways

**FIGURE 8:**
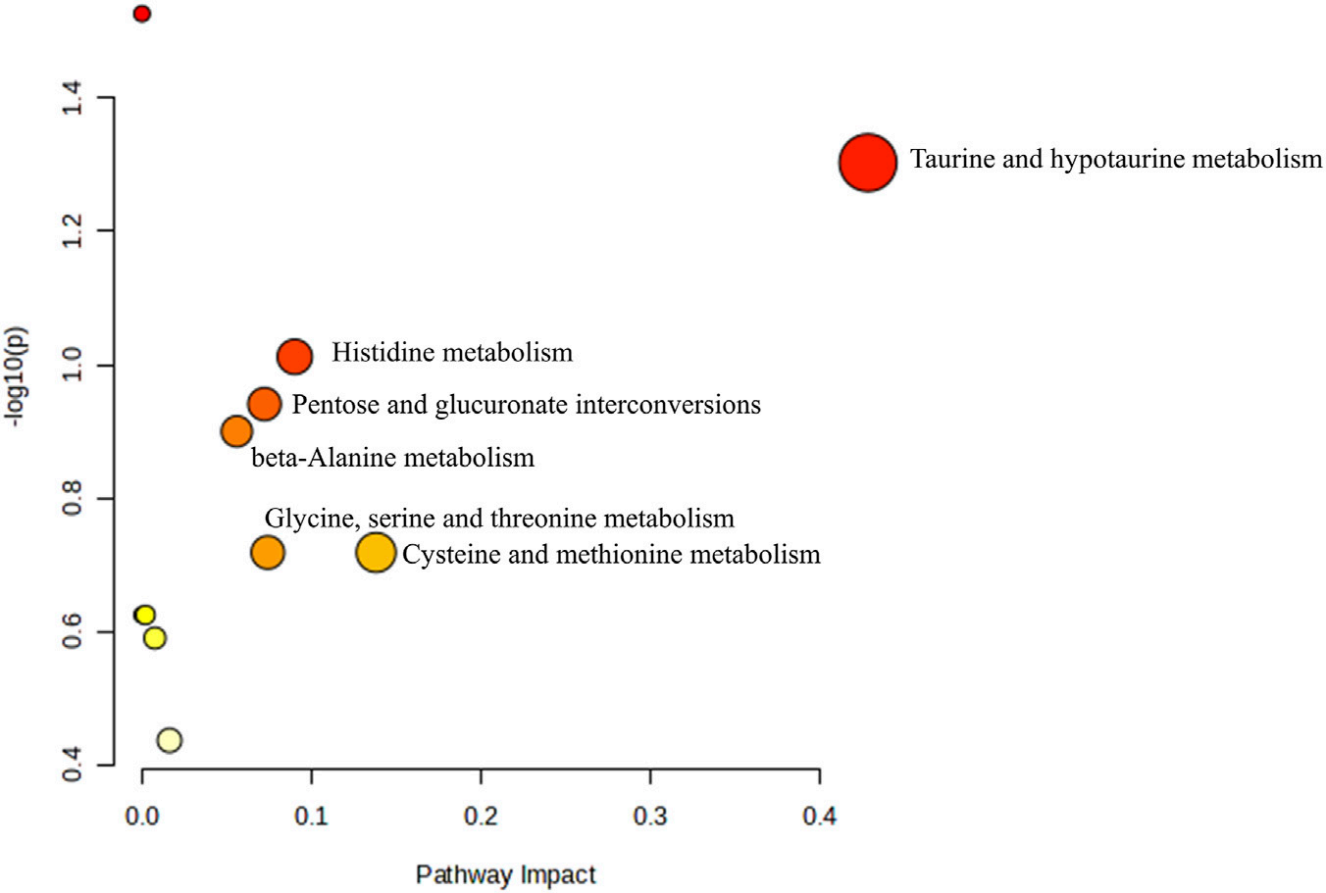
Analysis of potential metabolic pathways in RRTJ.

Metabolomics pathway analysis was conducted using MetaboAnalyst 6.0 to identify and visualize the potential metabolic pathways impacted by RRTJ. The identified 61 biomarkers were found to be involved in six metabolic pathways in which the pathway impact was greater than 0.05. The six pathways were taurine and hypotaurine metabolism, histidine metabolism, β-alanine metabolism, glycine, serine and threonine metabolism, pentose and glucuronate interconversions, and cysteine and methionine metabolism (Figure 8). These findings suggest a significant relationship between metabolite abnormalities and these metabolic pathways in arsenicosis patients. In particular, RRTJ may exert its effects through antioxidant, anti-inflammatory, and regulatory dysmethylation mechanisms, thereby alleviating arsenic poisoning.

## 4 Discussion

Based on untargeted metabolomics, this study analyzed the protective effect of RRTJ on metabolic disorders in arsenicosis patients, and initially explored the key metabolites and associated metabolic pathways involved in the potential mechanisms. The findings of the study demonstrate that RRTJ intervention has affected 61 differential metabolites. These metabolites were mainly enriched in six metabolic pathways, including taurine and hypotaurine metabolism, histidine metabolism, β-alanine metabolism, glycine, serine and threonine metabolism, pentose and glucuronate interconversions, and cysteine and methionine metabolism. These pathways are closely associated with antioxidant defence, anti-inflammatory responses, and the synthesis of methyl donors.

RRTJ exhibits a wide range of biological activities, mainly due to its high content of active compounds including flavonoids, vitamin C, polyphenols and polysaccharides (Jain et al., 2024). After these ingredients into the body, can adjust the variety of metabolites play a role of healthcare (Ji et al., 2022). Among them, polyphenols and flavonoids can significantly increase the content of glutathione (GSH) in plasma, and enhance the body’s antioxidant defense and immune capacity (Lapenna, 2023; Tang et al., 2023; Grabez et al., 2022). In addition, vitamin C and polyphenols may reduce plasma homocysteine (Hcy) levels by regulating the activity of enzymes in the methionine cycle (Ammar et al., 2020; Wu et al., 2024).

Studies have shown that individuals with arsenic poisoning exhibit severe oxidative and antioxidant imbalances (Hu et al., 2020). Enhancing antioxidant capacity is considered an effective approach to alleviate arsenic poisoning. RRT is a natural product known to have antioxidant capacity. On one hand, RRTJ can reduce the generation of ROS. Arsenic exposure leads to elevated levels of hypoxanthine, which induces oxidative stress by generating excessive ROS, resulting in cellular dysfunction and ultimately apoptosis or necrosis (Ryu et al., 2016; Biasibetti-Brendler et al., 2018). This process promotes multi-organ damage in arsenic poisoning (Kim et al., 2017; Toledo-Ibelles et al., 2021). Following RRTJ intervention, plasma hypoxanthine levels significantly decreased, thus diminishing ROS production. On the other hand, RRTJ also functions to scavenge ROS. L-carnosine levels are reduced in arsenic poisoning and can serve as a marker to distinguish the severity of arsenicosis (Zhang et al., 2023). L-carnosine contains numerous polyphenolic molecules that can scavenge ROS free radicals, thereby reducing malondialdehyde (MDA), superoxide dismutase (SOD), glutathione peroxidase (GPx), and catalase, and thus preventing cellular oxidative damage (Caruso et al., 2019; Park et al., 2022; Mousa and Aldebasi, 2021). In this study, RRTJ significantly increased the plasma concentration of L-carnosine, thereby promoting its ROS-scavenging and antioxidant effects. Additionally, we found that the levels of the oxidative stress biomarker prostaglandin F2-α and the antioxidant taurine were significantly reduced following RRTJ intervention (Nagui et al., 2022). Arsenic exposure induces oxidative stress, leading to a compensatory increase in taurine levels to exert antioxidant functions. After RRTJ intervention, oxidative stress in arsenicosis patients was partially alleviated, with levels of prostaglandin F2-α and taurine gradually decreasing.

Dysregulated inflammation responses is the crucial mechanisms that cause the health hazard of arsenism patients (Liu et al., 2022; Wang et al., 2021). In this study, we found that the effects of RRTJ on metabolites are closely associated with the regulation of imbalanced inflammatory responses. 2-isopropylmalate is a metabolite that protects cells from damage. Interestingly, our findings showed that RRTJ increased the concentration of 2-isopropylmalate in the plasma of arsenicosis patients. An increase in the 2-isopropylmalate content can inhibit the expression of the pro-inflammatory cytokines IL-6 and TNF-α, and the activation of NF-κB in cells, thereby exerting an anti-inflammatory effect (Bae et al., 2021; Choi et al., 2021). The other metabolite, D-xylose, is a product of the direct metabolism of xylitol by xylose reductase and the coenzyme NAD(P)+. The significant increase in D-xylose after the RRTJ intervention may further prevent injury and inflammation by stimulating the synthesis and secretion of proteoglycan (PG) and glycosaminoglycan (GAG) in cells and preventing the degradation of heparan sulphate (Cheudjeu, 2020). Together, these results suggest that RRTJ exerts an anti-inflammatory effect by increasing the contents of 2-isopropylmalate and D-xylose.

Arsenic can deplete S-adenosylmethionine (SAM) through As3MT, resulting in an insufficient supply of SAM. This blocks the methylation modification, disturbing the normal physiological function of genes, resulting in a toxic effect (Li et al., 2023). The glycine, serine and threonine cycle as well as the methionine cycle are closely related to one-carbon metabolism. The findings of the current study revealed a significant decrease in the plasma glycine level and a significant increase in the homocysteine level in arsenicosis patients. A reduction in the glycine level leads to decreased SAM synthesis while an elevation of the homocysteine level reflects increased SAM consumption. Both factors affect proper body methylation modification. Glycine contributes carbon units for one-carbon metabolism and participates in glutathione and SAM synthesis. Our previous *in vivo* experiments demonstrated that an RRTJ intervention can increase the SAM supply, regulate the H3K36me3 methylation level, and improve arsenic-induced liver injury in rats (Ma et al., 2023). By comparing the plasma glycine and homocysteine levels before and after the RRTJ intervention among arsenic poisoning patients, the current study observed a significant increase in the glycine content accompanied by a notable decrease in the homocysteine content. RRTJ intervention promotes an elevated SAM content by supplementing the body with glycine (Ling et al., 2022). Simultaneously, it binds to methyltransferases using SAM as a cofactor for protein, small-molecule, lipid and nucleic acid methylation to maintain normal physiological function within the body (Winter et al., 2022). The reduction in the homocysteine level following the RRTJ intervention in this study can be attributed to its conversion into SAM through binding with ingested methyl groups, thereby providing the body with necessary methyl donors (Mohammad and Kowluru, 2020). Therefore, it is speculated that RRTJ can restore arsenic-induced SAM deficiency by promoting glycine synthesis and reducing the homocysteine content in the body to alleviate arsenic-induced methylation disorder.

We have previously explored the antagonistic effects of RRTJ on immune imbalance, oxidative stress and methylation disorder induced by arsenic. On this basis, this study conducted an RRTJ intervention in arsenicosis patients, further supported the aforementioned viewpoints at the plasma metabolite level, and obtained 61 kinds of metabolites with possible effects such as L-carnosine, Dl-homocysteine, prostaglandin f2. α. This provides insights for subsequent research on the mechanism of alleviating arsenic poisoning from a metabolic perspective. Despite its strengths, this study still has some limitations. Firstly, this was an observational study, and the mechanism by which potential metabolites targeted by RRTJ to alleviate arsenicosis still need to be further verified in future *in vitro* experiments. Secondly, RRTJ contains a variety of active components, and subsequent research could explore which specific components play key roles in inducing changes in metabolites.

## 5 Conclusion

This study demonstrated that RRTJ can regulating metabolic disorder in arsenicosis patients. The restoration of 61 different metabolites may exert antioxidant, anti-inflammatory, and methylation donor synthesis functions through multiple metabolic pathways. These findings provide new insights into the potential mechanisms by which RRTJ may protect against arsenic poisoning.

## Data Availability

The raw data supporting the conclusions of this article will be made available by the authors, without undue reservation.
